# Evaluation of Factors Contributing to Diagnosis of Crohn’s Disease in the Face of Increasing Trend in Pakistan

**DOI:** 10.1093/crocol/otae015

**Published:** 2024-03-22

**Authors:** Tayyab Saeed Akhtar, Bilal Ashraf, Kanza Zahid, Sameen Abbas, Anosh Sana, Abdul Rauf Khan, Faiqa Ijaz, Faisal Riaz

**Affiliations:** Center for Liver and Digestive Diseases, Holy Family Hospital, Rawalpindi, Pakistan; Rawalpindi Medical University, Rawalpindi, Pakistan; Rawalpindi Medical University, Rawalpindi, Pakistan; Department of Pharmacy, Quaid-i-Azam University, Islamabad, Pakistan; Department of Pharmacy, Quaid-i-Azam University, Islamabad, Pakistan; Rawalpindi Medical University, Rawalpindi, Pakistan; Fatima Jinnah Medical University, Lahore, Pakistan; Rawalpindi Medical University, Rawalpindi, Pakistan

**Keywords:** Crohn’s disease, inflammatory bowel disease, diagnostic issues, LMICs (low and middle-income countries), health outcomes

## Abstract

**Background:**

Crohn’s disease (CD) is characterized by granulomatous inflammation of the digestive tract. Diagnosing CD involves assessing clinical symptoms, radiological and endoscopic findings, and histopathological evidence. Although previously considered a disease in developed countries, CD is increasing in developing nations, but challenges exist in diagnosing CD promptly. This study aims to report diagnostic parameters for early and correct CD diagnosis in Pakistan.

**Methodology:**

A retrospective analysis from June 2016 to August 2023 of 22 CD patients was done, by data from medical records, questionnaires completed at diagnosis, and telephonic interviews. Baseline demographic and clinical characteristics were assessed, and patients were categorized using the Montreal classification.

**Results:**

CD was diagnosed in 22 patients, with a 1:1 male-to-female ratio with a mean age of 33 years (range 15–55 years). Symptoms at presentation included abdominal pain (95.5%), watery diarrhea (86.4%), fever (31.8%), rectal bleeding (54.5%), and weight loss (81.8%) with 68% having symptoms for over 12 months before diagnosis. Disease characteristics were diverse, with various patterns of involvement and histopathological findings.

**Conclusions:**

In resource-limited countries like Pakistan, the timely diagnosis of CD presents a significant healthcare challenge. Therefore, it is necessary to tackle these complex problems by enhancing diagnostic capabilities, raising medical awareness, and improving access to healthcare resources.

## Graphical Abstract

**Graphical Abstract:**
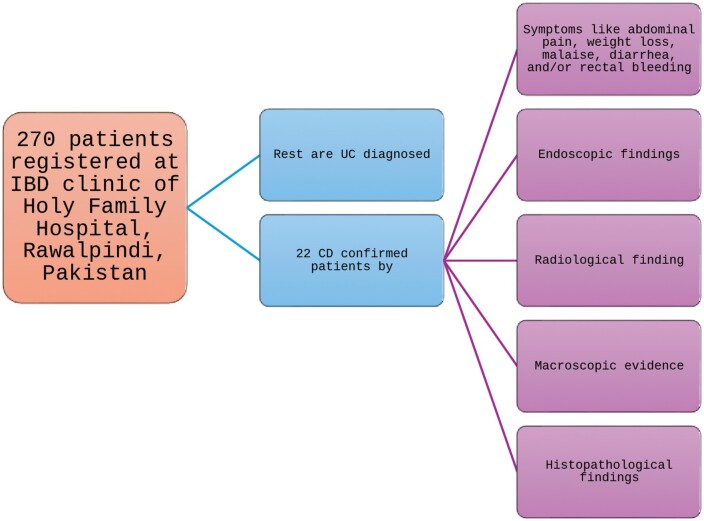

## Introduction

The term Inflammatory bowel disease (IBD) is used to describe a chronic relapsing, non-infectious disorder of the gastrointestinal tract, consisting of two subcategories: Ulcerative colitis (UC) and Crohn’s disease (CD). Although these disorders have been acknowledged for centuries, the precise mechanisms responsible for the pathophysiological basis of these conditions remain to be defined conclusively. The CD is believed to be secondary to a complex interplay of genetic influences, environmental triggers, and immune mechanisms, leading to a dysregulated innate and adaptive mucosal immune response to indigenous microbial flora and food antigens.^1^ In genetically predisposed individuals, environmental triggers such as drugs, toxins, infectious agents, and intestinal microbes may induce an atypical immune response. This results in inflammation of intestinal crypts which heralds the development of CD. Cryptitis, characterized by the inflammation of these crypts, progresses to ulceration of intestinal mucosa and submucosa. With further progression, transmural inflammation of the involved segment ensues, where inflamed regions are interspersed with areas of normal mucosa. This leads to the distinctive “skip lesions” observed in CD. The CD is characterized by focal asymmetric transmural granulomatous inflammation, which can involve any part of the gastrointestinal tract, from the mouth to the anal canal.

Key features to diagnose CD include clinical manifestations (abdominal pain, diarrhea, hematochezia, weight loss, perianal disease), laboratory (ESR, C-reactive protein, iron deficiency or vitamin B12 deficiency anemia, fecal calprotectin, anti-saccharomyces cerevisiae antibodies (ASCA), Anti-neutrophil cytoplasmic antibodies (p-ANCA), hypoalbuminemia), radiological (wall thickening and edema, deep ulcerations, strictures, fistulous tract formation) endoscopic (discontinuous “skip” lesions, aphthoid ulcers, longitudinal fissures/ulcers, cobblestone pattern), and histopathological findings (focal transmural inflammation, non-caseating granulomas, intraepithelial lymphocytosis).^2^

Although considered a disease of developed nations in the past, the prevalence of CD in developing nations of Asia is on the rise,^3^ possibly secondary to improved hygiene (hygiene hypothesis), changes in environmental factors, westernized diet, and socioeconomic status.^4^ A recently conducted study on the incidence of IBD in Asian countries in a population-based inception cohort from Korea showed a 40× increase in the incidence of CD during the past three decades.^5^ This change in trend is associated with misdiagnosis and mismanagement of patients with severe clinical implications affecting mortality and morbidity.

Substantial evidence-based epidemiological data with regard to the incidence and prevalence of CD is lacking in Pakistan. In the resource-constrained zone, the scarcity of essential diagnostic tools, coupled with the paucity of healthcare infrastructure and medical expertise, often impedes the timely identification of CD. Diagnosing Crohn’s disease can be challenging due to a wide network of different conditions that exhibit similar clinical presentations. These conditions include infectious colitis, ischemic colitis, drug-induced colitis, malabsorptive syndromes, irritable bowel syndrome, and gastrointestinal tuberculosis. Additionally, the low levels of awareness among patients in these areas add another layer of complexity, as delayed recognition and diagnosis significantly contribute to the worsening of outcomes. Insufficient healthcare resources and a potential lack of awareness among primary care physicians regarding the increasing prevalence of CD further compound the problem, resulting in delays in diagnosis and treatment. This is made worse by a lack of patient access to specialist gastroenterologist facilities in most regions of our country due to a lack of tertiary care hospitals. As there is no cure for CD, the financial burden of medications and surgical interventions required for managing CD and its complications adds another barrier in effectively managing these patients. So, we sought to report associated diagnostic parameters of Crohn’s disease which are needed for early and correct diagnosis as a tool of long-term management in Pakistan, a low-income country.

## Methodology

### Patients

**Figure 1.**
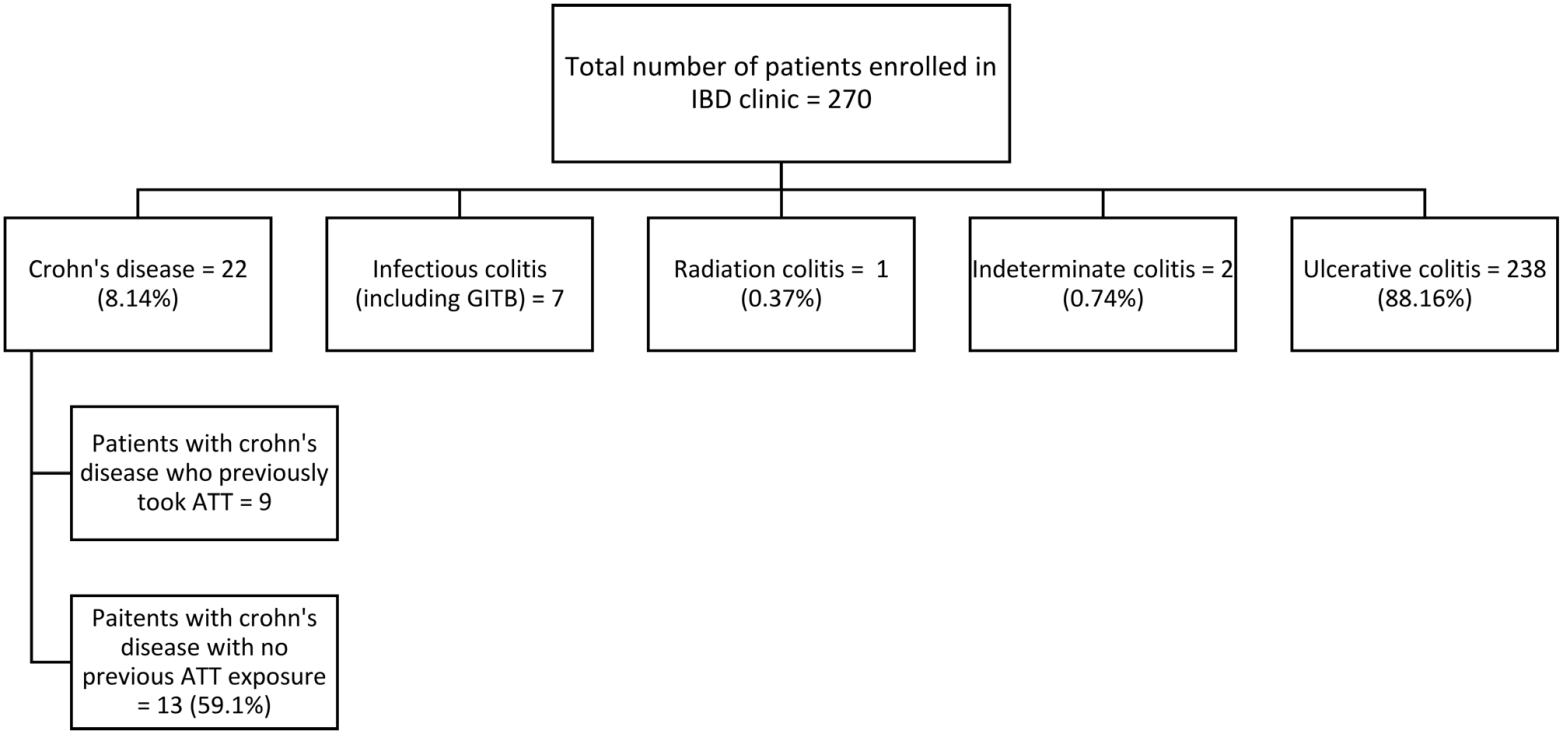
Consort chart showing the prevalence of cases at IBD clinic of Holy Family Hospital, Rawalpindi, Pakistan.

Between June 2016 and August 2023, a total of 270 patients were registered at the inflammatory bowel disease (IBD) clinic of Holy Family Hospital, Rawalpindi, Pakistan. Out of these cases, 22 patients were diagnosed and managed for Crohn’s disease as shown in Figure 1. To be diagnosed with CD, patients had to meet at least two of the following criteria for a minimum of 3 months:

- A history of abdominal pain, weight loss, malaise, diarrhea, and/or rectal bleeding.
- Endoscopic findings of mucosal cobblestoning, linear ulceration, skip lesions, or perianal disease.
- Radiological findings of stricture, fistula, mucosal cobble stoning, or ulceration.
- Macroscopic evidence of bowel wall induration, mesenteric lymphadenopathy, and “creeping fat” observed during laparotomy.
- Histopathology showing transmural inflammation and/or epithelioid granulomas.

Patients who initially could not be differentiated from tuberculosis (TB) and received standard anti-tuberculous medication were also included. However, individuals with indeterminate colitis were excluded from this study. Additional diagnostic procedures, such as colonoscopy, small bowel follow-through, or computed tomography, were performed based on the physician’s judgment. In cases of complications, such as perforation, infection, bleeding, or advanced flare-ups, patients were admitted to the ward and received appropriate treatment.

### Methods

Written informed consent was taken from all participants. Retrospective data analysis was done using information from telephonic interviews, diagnosis-related questionnaires, and healthcare records. We assessed baseline data on age, sex, the habit of smoking, familial history of inflammatory bowel disease (IBD), perianal disease history, the activity of the disease, location, and behavior. Patients were divided into subgroups based on the position and behavior of the disease via Montreal classification. Behavioral characteristics (B1, inflammatory, B2, stricturing, and B3, penetrating) and disease location (L1, small bowel, L2, colon, and L3, small bowel and colon) were noted. We assessed the rates of disease flare-ups, perianal disease, entero-colic/entero-vesical fistula, need for intestinal resection, and survival to look into the subsequent progression of CD.

### Statistics

Continuous variables were expressed as means with percentages. Statistical analysis was performed using SPSS for Windows, ver. 25.0 (SPSS Inc., Chicago, IL, USA).

## Results

Individuals with symptoms suggestive of CD (diarrhea, abdominal pain, weight loss, perianal disease) underwent endoscopic evaluation. A diagnosis was confirmed when distinctive features of CD, including linear ulcerations, skip lesions, cobblestone mucosa, and rectal sparing, were observed during ileocolonoscopy, and biopsies were taken for further characterization of the disease. In cases where endoscopy was inconclusive, patients underwent radiological assessment (CT/MRI abdomen & pelvis) to investigate alternative causes. Radiological tests were also performed in individuals diagnosed with CD who presented with CD-related complications (fistulas, intra-abdominal abscess, bowel wall thickening/strictures). Diagnosis of CD was made in 22 patients, 11 of whom were males. and 11 were females with a male-to-female ratio of 1:1 (Table 1). The mean age at diagnosis was 33 years with a range of 15–55 years. 22% had a positive family history of inflammatory bowel disease at the time of diagnosis. History significant for past or current tobacco smoking was found in 13.6% of patients. Upon preliminary workup, 86.4% were found to be anemic, 86% had elevated ESR, 54.5% had raised CRP, and 22.7% had hypoalbuminemia. Eight (36%) patients underwent emergency laparotomy due to acute abdomen. Of these eight, 3 (37.5%) had intestinal perforation, another 3 (37.5%) had intestinal obstruction, and while remaining 2 (25%) were operated on for diagnostic purposes. A sizeable majority of patients (68%) had symptoms for a protracted period of over 12 months before diagnosis of CD. Nine patients (40.9%) had previously received a course of anti-tuberculous medication due to clinical suspicion of gastrointestinal tuberculosis (Table 1).

**Table 1.** Demographic and clinical characteristics of patients.

Variables	*N*	%age
Male	11	50
Female	11	50
Family history of IBD	5	22.7
Intestinal obstruction	3	13.6
Intestinal perforation	3	13.6
Abdominal surgery	8	36.4
Enterocutaneous fistula	1	4.5
Appendectomy	4	18.2
Previously treated for gastrointestinal tuberculosis	9	40.9
Tobacco smoking	3	13.6
Anemia	19	86.4
Raised ESR	19	86.4
Raised CRP	12	54.5
Duration of symptoms		
Less than 6 months	3	13.6
Less than 12 months	4	18.2
More than 12 months	15	68.2
Frequency of Stools (per day)		
Less than 4	3	13.6
4–6	12	54.5
More than 6	7	31.8

**Figure 2.**
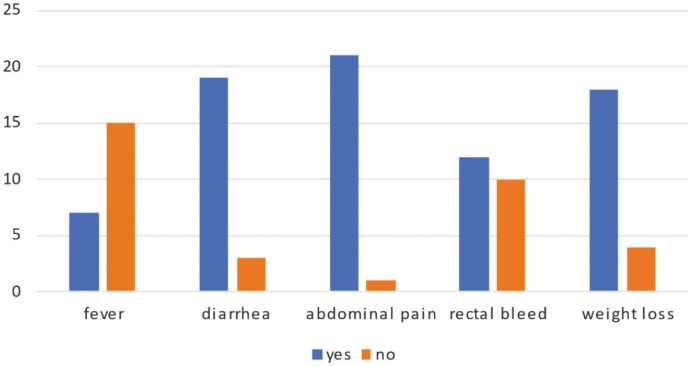
Primary symptoms at the time of presentation of CD patients (*N* = 22).

Primary symptoms at presentation were abdominal pain (95.5%), watery diarrhea (86.4%) with a bowel frequency of 4–6 times a day (54.5%), fever (31.8 %), rectal bleed (54.5%), and weight loss (81.8%) as depicted in Figure 2.

Patients were categorized based on Montreal classification summarized in Table 2. One patient (4.5%) had disease onset at or before 16 years of age (A1), another 15 (68.1%) had symptom onset between 17 and 40 years of age (A2), while the remaining six (27.2%) had onset after 40 years of age (A3). Thirteen individuals (59%) had predominant colonic involvement (L2), another eight individuals (36.4%) had concomitant colonic and ileal involvement (L3), with only one patient (4.5%) had ileal involvement without colonic involvement (L1). Disease behavior was classified into six subcategories based on predominant disease behavior and perianal involvement (p) as a modifier. seven patients (31.8%) had a non-penetrating, non-stricturing pattern without perianal involvement (B1) while another three individuals (13.63%) had non-penetrating, non-stricturing disease with perianal involvement (B1p). Predominantly stricturing phenotype was found in 10 patients (45.45%), of which 5 (22.7%) had no perianal involvement (B2) while the remaining five (22.7%) had perianal involvement (B2p). two patients (9.9%) had predominantly penetrating disease patterns with no perianal involvement (B3).

**Table 2.** Characteristics by Montreal classification of CD patients (*N* = 22).

Variables	*N*	%age
Age of onset		
A1 <16 years	1	4.5
A2 <17–40 years	1*5*	68.1
A3 >40 years	6	27.2
Location		
L1: Terminal ileum	1	4.5
L2: Colon	13	59
L3: Ileo-colon	8	36.3
L4: Upper GIT	0	0
Behavior		
B1: Non-stricturing, non-penetrating	7	31.8
B1p: Non-stricturing, non-penetrating with perianal involvement	3	13.6
B2: Stricturing disease	5	22.7
B2p: Stricturing with perianal involvement	5	22.7
B3: Penetrating disease	2	9.9
B3p: Penetrating with perianal involvement	0	0

The majority of the patients had findings suggestive of gut inflammation with 17 (77%) having abdominal lymphadenopathy with 11 (50%) having peri-colonic fat stranding. Thickening of the intestinal wall was the predominant finding in 15 (68.2%) patients. Abdominopelvic ascites was seen in 7 (32%) patients. One patient had an entero-enteric and an enterocutaneous fistula, while another patient had intra-abdominal abscess formation. Most patients (95.4%) had histopathology predominantly characterized by chronic mucosal inflammation, of which 10 (45%) had right-sided colitis, five patients (22.7%) had left-sided colitis while another 5 (22.7%) had pancolitis. one patient had isolated ileal inflammation with no active colitis. A total of 4 (18%) patients had non-caseating granulomas. Crypt abscess was seen in five patients (22.7%) while distortion of crypt architecture was seen in 2 (9.1%) patients. Dysplasia or Paneth cell metaplasia was not seen in any participant. Endoscopic and histopathological findings are summarized in Tables 3 and 4, respectively.

**Table 3.** Endoscopic examination findings of CD patients (*N* = 22).

Variables	*N*	%age
Ulcers typical of Crohn’s disease	19	86
skip lesions	16	72.7
Mucosal appearance		
Normal	1	4.*5*
Friable, erythematous	14	66
Cobblestone	*5*	22.7
Ulcerated	1	4.*5*
Gangrenous	1	4.*5*
Stricture		
Ileum	3	13.6
Ascending colon	1	4.*5*
Transverse colon	1	4.*5*
Sigmoid colon	2	9.1
Rectosigmoid junction	1	4.*5*
Rectum	2	9.1
Ileal involvement	9	40.9
Large intestine involvement		
Cecum	*5*	22.7
Right-sided colon	*5*	22.7
Transverse colon	1	4.*5*
Left sided	*5*	22.7
Generalized	*5*	22.7
Rectal sparing	14	63. 6
Colitis		
Right sided	10	45*.4*
Left sided	4	18
Pancolitis	7	31.8

**Table 4.** Histopathological examination findings of CD patients (*N* = 22).

Variables	*N*	%age
Granulomas	4	18
Ileum	1	4.5
Cecum	2	9.1
Sigmoid colon	1	4.5
Pseudo polyps	12	54.5
Crypt abscess	5	22.7
Crypt distortion	2	9.1
Dysplasia	1	4.5

## Discussion

With increasing technological advancement and expeditious industrialization among the countries of South Asia, many conditions previously considered rare are being seen at an increased rate. One such condition is IBD, a chronic inflammatory disease of the gastrointestinal tract with a relapsing and remitting natural history. IBD comprises two distinct entities: CD and UC. CD has been recognized as a disease entity since 1932, yet the precise mechanism leading to its pathogenesis remains to be defined. The CD is an autoimmune granulomatous condition of the gastrointestinal tract which can involve any part of the alimentary tract but commonly affects the terminal ileum and large intestine.

During the 20th century, IBD was mainly a disease in westernized countries of North America and Europe.^6^ At the beginning of the 21st century, IBD spread rapidly throughout Southeast Asia where resources were already scarce.^7^ Due to financial constraints, a huge population burden, and fewer hospitals coupled with an extreme lack of awareness and acceptance, timely diagnosing CD imposes a massive challenge on our healthcare system. Furthermore, diagnosing CD can be quite challenging due to a wide network of different conditions that exhibit similar clinical presentations. These conditions include infectious colitis, ischemic colitis, drug-induced colitis, malabsorptive syndromes, irritable bowel syndrome, and gastrointestinal tuberculosis. The most commonly reported provider challenge is differentiating between CD and intestinal tuberculosis (ITB), due to the high prevalence of TB in lower middle-income countries and its overlap of symptoms and endoscopic features.^8^ This underscores the necessity for distinguishing CD from other resembling conditions and the challenges faced by medical practitioners for appropriate treatment in developing countries.

As per our inclusion criteria, abdominal pain was reported in 95.5%, watery diarrhea in 86.4%, per rectal bleed in 54.5%, and weight loss in 81.8% of people. These are considered hallmark symptoms of CD in the medical literature.^9^ The majority of patients in our cohort had these symptoms for 12 months or longer prior to being diagnosed with CD. A delay in diagnosis of CD may lead to a decrease in quality of life, increased healthcare costs, higher rates of penetrative/fistulizing phenotype, and an increase in the need for surgical intervention.

Skip lesions were found in 72% of patients, 22% had cobblestone mucosa while 36% had perianal involvement (27% had perianal fistula, 4.5% with perianal ulcer, and 4.5% with perianal skin tags). Among the radiological findings suggestive of CD, stricture formation was seen in 45% of individuals. Non-caseating granulomatous inflammation was seen in 18.18% of patients while none of the patients had creeping fat. One in every three patients who presented to our clinic was previously treated with ATT due to clinical suspicion of gastrointestinal tuberculosis and 55% of these had intestinal stricture at the time of diagnosis of CD. This can be attributed to a combination of delay in diagnosis with disease progression and a direct effect of anti-tuberculous therapy. Presumptive use of ATT can lead to early stricture formation in CD patients.^10^ Furthermore, the use of ATT in patients with CD may lead to an increased need for surgical interventions.^11^ Early recognition and treatment of CD may lead to favorable outcomes for these groups of patients.

With regard to non-invasive diagnostic procedures, the best-considered markers are serum CRP and fecal calprotectin.^2^ The preferred inflammatory marker is CRP because it relates better with the endoscopic activity of the disease in CD and is more sensitive than the erythrocyte sedimentation rate (ESR) for the assessment of acute abdominal discomfort among individuals with IBD.^12^ CRP levels are higher in active CD than in UC, and its sensitivity can vary from 70% to 100% in the potential diagnosis of CD compared to irritable bowel syndrome.^13^ Our results demonstrated a raised CRP in 54.5% of patients which is consistent with the above data regarding CRP. 95% did not have fecal calprotectin levels assessed, primarily due to non-affordability of the patients and non-availability of these tests in most regions of our country. Pooled sensitivity and specificity of fecal calprotectin are 93% and 96%, respectively with a diagnostic accuracy higher for CD (sensitivity, 83%; specificity, 85%) than for UC.^14^ Although CRP has a higher sensitivity in diagnosing patients with CD, it lacks specificity when used alone. CRP is an acute phase reactant protein, produced by the liver in inflammatory conditions. Thus, its use in isolation does not accurately predict the presence of CD. Use of fecal calprotectin with serum CRP may complement one another, providing a higher degree of evidence in favor of CD.

Adding another layer, more than 1/3 of general physicians lack confidence in identifying the key symptoms of CD.^15^ A validation study discovered that the sensitivity and specificity of an index based on a questionnaire created by the International Organization for IBD on symptoms as well as signs alone were only 50% and 58%, respectively. However, the degree of sensitivity and accuracy significantly increased when combined with fecal calprotectin, a verified non-intrusive biomarker identification of inflammation in the intestines.^16^ Where invasive procedures such as biopsy and endoscopy are unavailable, incorporating the diagnostic pathways using fecal calprotectin in the primary care setting has demonstrated value in assisting healthcare providers in evaluating the risk, which ultimately improves the time to screening and results in resource and cost savings.^17^ This may be helpful in our primary care setups where people do not have financial help or access to advanced diagnostic procedures. Hence, CRP and fecal calprotectin levels can be used as screening tools in patients with high clinical suspicion of IBD at the primary care level so that patients can be referred to a specialist early in the disease process.

9**5**% of our patients did not have their anti-saccharomyces cerevisiae antibody (ASCA) antibodies tested as a part of their serological investigations due to limited availability and a lack of affordability. A meta-analysis found that the pooled sensitivity, specificity, and diagnostic accuracy of ASCA in the diagnosis of CD was 33%, 83%, and 57%, respectively.^10^ The significance of ASCA in the evaluation of CD remains to be emphasized worldwide. Additional research is needed to establish the diagnostic utility and cost-effectiveness of ASCA, more precisely in evaluating individuals suspected of having CD in developing nations.

In terms of disease location, 45% of cases had involvement of the right part of the large intestine, and 36% involved both the large intestine and the ileum at the same time. The fact that ileocolonic disease also constitutes the most prevalent CD phenotype in other Asian nations like Japan (65.8%), as well as South Korea (77.7%) makes this information significant.^18^ Clinical relevance exists for this disease behavior because it is linked to the emergence of CD-related adverse effects and the requirement for surgery. In roughly 6%–16% of cases, CD can present with severe complications requiring urgent surgery^13^ while in our case, 36% first presented to the emergency department with an acute abdomen. This may in part be due to a smaller sample size of our study, geographical variation in disease phenotype and presentation, diagnostic delay, mismanagement of patients, or a combination of these. Further studies on the acute presentation of CD are required in our setup to better elucidate these differences.

In summary, the lack of specialist gastroenterologists in rural areas and a high threshold among primary care physicians collectively contribute to a delayed diagnosis, and potentially, overlooking cases of CD. Moreover, this high threshold of suspicion results in the underutilization of already limited non-invasive tests like CRP and fecal calprotectin. Educating healthcare professionals, particularly those working in primary care, through workshops, seminars, and continuing medical education programs is crucial to address this issue. Additionally, raising awareness about CD among the general public through brochures, pamphlets, school and community programs, and media engagement could aid in early patient presentation to healthcare facilities. Furthermore, enhancing the availability of non-invasive tests such as CRP and fecal calprotectin and invasive tests including endoscopy facilities in remote areas may further aid in early patient diagnosis.

## Future Prospects

The incidence/prevalence of CD is increasing in Southeast Asia where the resources are already limited. Prompt diagnosis and initiation of therapy for CD may be associated with improved outcomes for patients, and a lower healthcare cost overall. Further studies are required in our setup to better define the epidemiological pattern of CD in Pakistan. Additional research is required to evaluate the role of laboratory tests and their diagnostic accuracy in our population. Moreover, educating medical practitioners on the increasing trends of CD and a lower threshold of suspicion is required for timely referral to a gastroenterologist. We are of the opinion that our research can serve as a cornerstone for broader investigations, manifestation, and therapeutic options for patients with CD with the ultimate goal of enhancing the well-being of individuals affected by this condition.

## Limitations

Our study was based on the patients presenting to the IBD clinic of Holy Family Hospital, mostly referred from the periphery and underdeveloped areas of Punjab, so the results do not apply uniformly to all the patients as compared if it were a population-based study. Also, the CD is new to our population and the physicians as well, basic health care units and rural health centers do not have the expertise to timely diagnose the patients coupled with the fact that most are being treated along the lines of gastroenteritis and Gastrointestinal Tuberculosis. Lack of response and increase in severity of symptoms compel patients to visit a tertiary health care system as in our case, Holy Family Hospital. This may have resulted in a poor prognosis for our chosen population.

## Conclusions

The CD is characterized by focal asymmetric transmural granulomatous inflammation involving any part of the GI tract. Accurate identification of Crohn’s disease requires a well-coordinated healthcare infrastructure equipped with essential facilities such as proficient gastroenterologists, colonoscopy, histopathology services, monitoring disease severity through fecal calprotectin, and access to immunosuppressive medications and biologics, complemented by surgical expertise. The health care of Pakistan constitutes a public and a private sector, and a very small amount of its GDB is spent on healthcare along with unfair distribution of funds, inadequate number of trained healthcare staff, and less focus on preventive health has been a major barrier to the provision of an effective service. Thus, timely diagnosing of CD imposes a massive challenge on the healthcare system in Pakistan. Consequently, addressing these multifaceted issues through improved diagnostic capabilities, increased medical awareness, and enhanced access to healthcare resources is paramount to mitigating the impact of CD in developing nations and ultimately improving patient outcomes.

## Data Availability

The original contributions presented in the study are included in the article, further inquiries can be directed to the corresponding author.
